# Modality-Specific and Multimodal ‘Associative’ Forms of Face and Voice Recognition Disorders in Patients with Right Anterior Temporal Lesions: A Review of Single-Case Studies

**DOI:** 10.3390/brainsci15121309

**Published:** 2025-12-04

**Authors:** Guido Gainotti

**Affiliations:** Institute of Neurology, Università Cattolica del Sacro Cuore, Fondazione Policlinico A. Gemelli, Istituto di Ricovero e Cura a Carattere Scientifico, 00168 Rome, Italy; guido.gainotti@unicatt.it; Tel.: +39-06-30156435

**Keywords:** face and voice recogniton disorders, associative prosopagnosia and phonagnosia, right anterior temporal lobes, non-verbal semantic disorders

## Abstract

**Introduction**: ‘Associative prosopagnosia’ and ‘associative phonagnosia’ are high-level post-perceptual face and voice recognition defects due to right anterior temporal lesions, but the relations between these two disorders are uncertain. It is, indeed, not clear if face and voice recognition disorders observed in these patients must be considered as independent modality-specific recognition defects or as fragments of a more general semantic disorder concerning the multimodal representation of known persons. **Aims of this study:** In this review, the relations between associative forms of face and voice recognition disorders were investigated in all patients with right anterior temporal lesions reported in the literature. A prevalence of ‘pure’ (modality-specific) forms could indicate that these are independent, modality-specific recognition defects, whereas a high frequency of voice- and face-associated disorders could suggest that they are components of a multimodal semantic disruption. **Results**: Results show that ‘associative prosopagnosia’ and ‘associative phonagnosia’ are observed sometimes as ‘pure’ forms, other times as associations between verbal and non-verbal defects of person recognition, and still other times as associations restricted to the non-verbal (face and voice) modalities of person recognition. Furthermore, in a patient with a multimodal face and voice recognition disorder, the lesion involved the right temporal pole, considered as the locus of convergence of face and voice recognition modalities. **Discussion**: These data suggest that specific lesions of the right anterior temporal lobes can disrupt the highest modality-specific levels of face and voice representations, whereas other equally selective lesions can disrupt the locus of convergence of face and voice recognition modalities.

## 1. Introduction

Face recognition disorders (‘prosopagnosia’) and, in particular, their ‘post-perceptual’ variants (‘associative prosopagnosia’) are systematically included in the most typical disabilities shown by patients affected by lesions of the anterior temporal lobes (ATLs). In particular, they are very frequent in patients with a right variant of semantic dementia and are considered as ‘markers’ of this disease (e.g., [1,2,3,4,5,6,7,8,9,10,11,12,13]). The nature of these post-perceptual disorders is, however, not very clear, because they are part of a complex multimodal recognition system, which takes input from perceptual (face and voice) and linguistic (proper name) modalities to identify people known to the subject.

### 1.1. The Nature of ‘Post-Perceptual’ Face Recognition Disorders

Different models have been proposed to explain how the process of person identification is achieved within each recognition modality, how these modalities interact, and how the semantic (biographic) information characterizing each person is accessed and organized. 

According to the first influential model of face recognition, proposed by Bruce and Young [14], the input of some lower-level perceptual processes, mapped onto specific recognition units for faces (FRUs) and voices (VRUs), converges into person identity nodes (PINs), allowing access to the semantic information of the corresponding person. Drawing on this line of thought, Ellis et al. [15] postulated the existence of two processing modules for faces and voices, working in parallel and including three steps. The first step should allow perceptual processes of visual and auditory “structural encoding”. The second step should be characterized by the activation of modality-specific “recognition units” for face (FRUs) and voice (VRUs), where the corresponding familiarity feelings should be generated. The final step should lead to the convergence of these two pathways into a “person identity node” (PIN), storing semantic information about the identified person. The problem raised by these models for the interpretation of pathological data consists in evaluating if face and voice recognition disorders observed in patients with lesions of the ATLs must be considered as independent modality-specific recognition defects or as fragments of a more general semantic disorder concerning the multimodal representation of known persons.

### 1.2. Structural Architecture of the Person Recognition System

From the anatomical point of view, the sequence of face- and voice-processing steps included in the models of person recognition proposed by Bruce and Young [14] and by Ellis et al. [15] begins in the fusiform and in the occipital face areas for faces (e.g., [16,17,18]) and in the superior temporal gyrus/sulcus for voices (e.g., [19]). These cortical areas carry out the visual and acoustical encoding necessary to establish face- and voice-specific representations, which are stored for both modalities in the anterior parts of the anterior temporal lobes (e.g., [20,21,22]). Within this general model, several authors have distinguished the verbal (proper name) from the non-verbal (face and voice) modalities of person recognition on the basis of theoretical and empirical reasons. The most important theoretical reason is that proper names, due to their strong links to language networks, are primarily integrated into the left hemisphere verbal knowledge, whereas faces and voices, due to their connections with visual, auditory, and socioemotional networks, are mainly integrated into the right hemisphere non-verbal semantic knowledge [23]. From the empirical point of view, the links between the right hemisphere and the non-verbal modalities of person recognition have been confirmed by anatomo-clinical and experimental investigations, which have shown the following:

(a) The cortical areas subserving the perceptual encoding of face and voice stimuli are more represented in the right than in the left hemisphere (e.g., [20,24,25,26,27,28,29]).
(b) A leading role of the right hemisphere in the generation of face and voice familiarity feelings is present both in normal subjects and in brain-damaged patients (e.g., [30,31,32,33,34,35]).
(c) Both face (e.g., [1,7,10,11,36,37,38,39,40]) and voice recognition disorders (e.g., [41,42,43]) are strongly associated with right hemispheric lesions.

**Figure 1:**
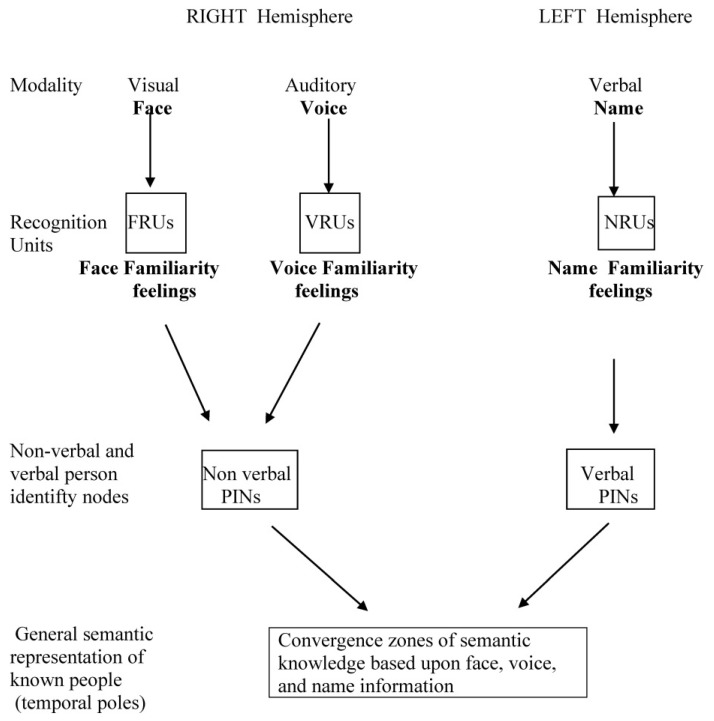
Figure caption.

The interactions between the steps included in general models of person recognition and the different contributions given by the right and the left hemispheres to the different channels of person recognition have been synthetically represented in Figure 1.

Data reported in Table 1 show that the right hemisphere mainly contributes (1) to the processing in specific recognition units of face and voice stimuli, (2) to the generation of the corresponding familiarity feelings, and (3) to the construction of non-verbal person identity representations, based on the convergence of this information. These data also show that the left hemisphere allows a similar processing of the verbal (proper name) stimuli and that the convergence of information stored in the verbal and non-verbal personal identity nodes leads to the construction of general semantic representations of known people.

### 1.3. Disorders of Person Recognition That Should Be Taken into Account to Clarify the Nature of Post-Perceptual Associative Variants of Prosopagnosia

To evaluate if associative forms of prosopagnosia must be considered as independent modality-specific recognition defects or as fragments of a multimodal semantic disorder, it is necessary to assess if the other post-perceptual modalities of person recognition tend to be disrupted in association with those concerning face recognition. However, to do this, it can be useful to take into account the historical evolution and our present knowledge of person recognition disorders. This process has drawn on two important distinctions concerning (a) the (face or voice) modalities of person recognition disrupted by the lesion and (b) the earliest (perceptual) or the most advanced (semantic) stages of the recognition process affected by the lesion. The term “prosopagnosia” (“prósōpon” = “face”) was originally coined by Bodamer [44] to designate the inability to recognize the face of known people, whereas the term “phonagnosia” (“phon” = “voice”) was proposed by Van Lancker and Canter [41] to designate a disorder of familiar voice recognition. The properly perceptual aspects of these recognition disorders have been labeled with the term ‘apperceptive disturbances’, whereas the most ‘cognitive’ disorders have been designated with the term ‘associative’ disturbances by Barton and Corrow [45]. From the clinical point of view, the distinction between ‘apperceptive’ and ‘associative’ forms of person recognition disorders is usually based on simple diagnostic criteria. Person recognition defects are, indeed, considered as ‘apperceptive’ when patients are impaired on face-matching tasks (or voice-matching tasks) of unfamiliar people, i.e., on tasks without a mnesic component. They are, on the contrary, considered as ‘associative’ if patients are unable to single out the ‘familiar’ faces or voices, discerning them from unfamiliar stimuli, and to retrieve the semantic (biographic) information that allows for identifying these familiar people.

### 1.4. Why a Parallel Study of Face and Voice Recognition Disorders Has Rarely Been Made

From the historical point of view, the first and the most important investigations in this area of inquiry have concerned both the apperceptive and the associative forms of prosopagnosia, whereas less interest has been devoted to the homologous forms of phonoagnosia. Even more neglected have been the possible relations between the associative forms of prosopagnosia and of phonagnosia, which constitute the target of the present review. The greater interest shown by investigators for the study of prosopagnosia (in comparison to that of phonagnosia) can be due to two main reasons. The first resides in the greater difficulty shown by both normal subjects and brain-damaged patients in processing voice in comparison with face stimuli (e.g., [46,47]). The second reason consists of the fact that there are many well-validated tests of face discrimination and recognition, a smaller number of tests comparing person recognition through both face and name (e.g., [1,48,49,50,51]), and few well-validated tests of voice discrimination and recognition. This asymmetry between the assessment of face and voice recognition disorders has led to different conclusions about the nature of associative prosopagnosia. Some authors (e.g., [52]) have, in fact, claimed that face recognition disorders observed in patients with right ATL lesions should be considered modality-specific forms of prosopagnosia, because in them, both face perception and retrieval of personal semantic knowledge from names are spared. Other authors (e.g., [53]) have, however, objected that not all these patients can be considered as instances of ‘associative prosopagnosia’, because, at least in some of them, the inability to recognize familiar people extends to voices and, to a lesser extent, to personal names. Liu et al. [40] developed this discussion in a well-documented study of face and voice perception and identification in 10 subjects with face recognition disorders acquired after cerebral lesions. They showed that a right ATL damage can cause a modality-specific associative prosopagnosia, whereas a co-occurrence of associative forms of prosopagnosia and phonagnosia was found only in two subjects with bilateral ATL lesions. The authors maintained that these results can be consistent both with the co-occurrence of independent associative deficits for face and voice and with a single multimodal semantic disorder. It could be noted, however, that the fact that this association was present only in patients with bilateral ATL lesions does not exclude that a multimodal semantic disorder restricted to the non-verbal ‘face’ and ‘voice’ modalities might be observed in patients with lesions restricted to the right anterior temporal lobe. This possibility is theoretically relevant with respect to models (e.g., [1,2,3]), which assume that semantic knowledge may be represented in a purely non-verbal format in the right ATL, because the concomitance of ‘associative’ forms of prosopagnosia and phonagnosia in patients with lesions restricted to the right should be consistent with this model.

### 1.5. Aims of the Present Review

Since several authors (e.g., [10,54,55]) have acknowledged that a major obstacle to the clarification of problems concerning the nature of prosopagnosia is the rarity of patients showing this disorder, I thought that a systematic review of all the single-case studies of associative prosopagnosia or phonagnosia reported in the literature could increase our knowledge of this debated issue. This review should be characterized—from the neuropsychological point of view, by an associative form of face or voice recognition disorder and—from the neuropathological point of view, by a lesion restricted to (or mainly involving) the right ATL. The points that I aimed to clarify, simultaneously taking into account the associative forms of prosopagnosia and of phonagnosia, were the following:

(a) Were subjects with ‘pure’ (i.e., modality-specific) forms of associative prosopagnosia or phonagnosia present in the surveyed patients?
(b) Were these ‘pure’ forms a sizeable part or a small minority of the total number of patients included in the review?
(c) Was the incidence of associative forms of prosopagnosia and of phonagnosia roughly similar, or was one of these defects clearly prevalent?
(d) Did face and voice recognition disorders habitually co-occur in the same patients, or were they usually observed in different patients?
(e) When face and voice recognition disorders co-occurred, was the sequence of their presentation randomly distributed, or did one of them usually appear first?
(f) Could the assessment of name recognition disorders help to clarify the meaning of the co-occurrence of associative forms of prosopagnosia and phonagnosia?

## 2. Methods

To clarify the above-mentioned points, I started with an explicit operational definition of the ‘modality-specific’ and of the ‘multimodal purely non-verbal’ and ‘multimodal general’ forms of face and voice recognition disorders investigated in this review. The following operational criteria were provided:

(a) Modality-specific associative prosopagnosia was defined as a disorder of people recognition selectively affecting familiar faces (but not the perceptual discrimination of unknown faces or the recognition of familiar voices or personal names).
(b) Modality-specific associative phonagnosia was defined as a disorder of people recognition selectively affecting familiar voices (but not the perceptual discrimination of unknown voices or the recognition of familiar faces or familiar names).
(c) Multimodal purely non-verbal semantic disorder of familiar people recognition was defined as a disorder of familiar people recognition through face and voice, but not through personal name.
(d) Multimodal general semantic disorder of familiar people recognition was defined as a disorder of familiar people recognition through face, voice, and personal name.

On the basis of these operational definitions, I took into account all publications that reported single-case studies of patients showing, from the neuropsychological viewpoint, an ‘associative’ disorder of face (prosopagnosia) or voice (phonagnosia) recognition and, from the neuroanatomical point of view, a right ATL lesion. With this aim in mind, I used PubMed and Web of Science to search for studies that included case reports relevant to this issue. The search keywords included terms related to disorders of face and voice recognition disorders (‘face recognition disorders’ OR ‘voice recognition disorders’ OR ‘associative prosopagnosia’) AND keywords related to the neuroanatomical lesions (‘right anterior temporal lesions’). The Boolean search strings were the following: “Face recognition disorders” AND “right anterior temporal lesions”; “Voice recognition disorders” AND “right anterior temporal lesion”; “Associative prosopagnosia” AND “right anterior temporal lesion”; “Associative phonagnosia” AND “right anterior temporal lesion”. The search timeframe period was between October 2024 and March 2025.

The flow diagram of the review process is the following:

In the Identification Stage, 74 records were identified from PubMed and/or Web of Science and from the references of the obtained articles. However, since the number of these studies was greatly unbalanced toward the associative forms of prosopagnosia in Screening Stage I, not only investigations that had objectively assessed face and voice recognition disorders but also case reports that had exhaustively assessed prosopagnosia but only clinically evaluated voice recognition disorders were included in this review. Explicit inclusion criterion was, therefore, the presence of an assessment (clinical or clinical and neuropsychological) of both face and voice recognition disorders, whereas explicit exclusion criterion was the lack of an assessment (clinical or neuropsychological) of both face and voice recognition disorders. This decision was due not only to the need of surveying a balanced number of patients with associative forms of prosopagnosia and phonagnosia, but also to the decision of evaluating the temporal order of presentation of face and voice recognition disorders when they were present in the same patients. Information on the temporal evolution of a cognitive disorder is, indeed, usually present more in (older) clinical papers than in more recent, controlled neuropsychological investigations. On the other hand, in this review were included not only patients reported as single-case studies but also patients reported in some detail within multiple single-case studies.

On the basis of these criteria, 51 of the previously identified papers were removed because (a) they were duplicates (N = 21), (c) concerned patients with only face recognition disorders (N = 22), (d) had not included a detailed description of single patients (N = 5), or for reasons concerning the poor neuroimaging data (N = 3). The remaining 23 publications reported data about the 22 patients who satisfied our inclusion criteria. While face recognition disorders had been formally tested in all these patients, in 5 of them (patients 4, 5, 7, 10, and 11), voice recognition disorders had been only clinically assessed.

**Figure 2:**
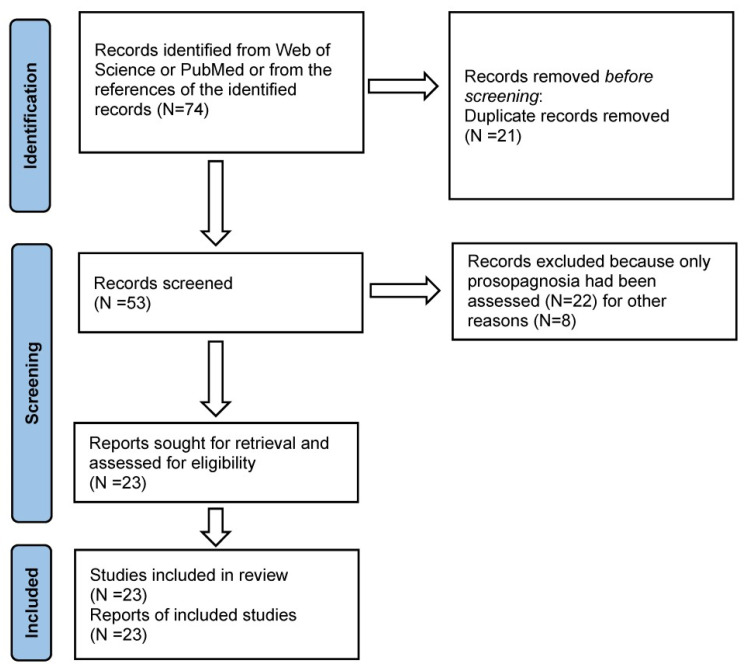
PRISMA 2020 flow diagram for patients reported in this review.

A PRISMA-style flow diagram has been included to summarize the selection process in Figure 2.

### Data Taken into Account for Each Patient Included in the Present Review and Tests Used to Formally Evaluate Face and Voice Recognition Disorders

The following data were taken into account (when available) for each of the 22 patients included in the present review:

(a) The etiopathological diagnosis.
(b) The main clinical data, including the onset of all symptoms and specifically of person recognition disorders, the relevant concomitant symptomatology, and their temporal evolution.
(c) The documented anatomical lesions.
(d) The face and voice recognition disorders.

The formal assessment of discrimination, familiarity, and identification components of these last defects had usually been taken separately into account for each patient, but different tests had been used by various authors to evaluate these forms of face and voice recognition disorders. The tests of **face processing** used in these studies included, among others, the following: (a) for face discrimination disorders, the Benton Face Recognition Test [56] and the face discrimination section of the Famous People Recognition Battery [57]; (b) for the evaluation of face familiarity and of face identification, the Warrington Recognition Memory Test [58], the Cambridge Face Memory Test [59], and the face familiarity and face identification sections of the Famous People Recognition Battery [57]; and (c) for the study of short-term familiarity with anonymous faces, the Famous Faces Test [60].

On the other hand, the assessment of **voice processing** included, among others, (a) for the evaluation of voice discrimination, the Voice Discrimination Test of Kreiman and Papkun [61] and the voice discrimination section of the Famous People Recognition Battery [57], and (b) for the evaluation of voice familiarity and voice identification, the Famous Voices Test [62], the Familiar Voice Recognition Test [63], and the voice familiarity and voice identification sections of the Famous People Recognition Battery [57].

(e) **Name recognition disorders** were also recorded, when available, in the selected papers to clarify the meaning of associations between face and voice recognition disorders.

## 3. Results

All the data taken into account in this review to clarify the nature of defects described under the heading of ‘associative prosopagnosia’ have been synthetically reported in Table 1.

**Table 1:** A systematic review of all single-case studies of patients with right anterior temporal lesions in which both face and voice recognition disorders have been assessed.

1, PV	([64]; [65])
Diagnosis	Herpes simplex encephalitis.
Main clinical data	Subjective complaints of inability to identify familiar people by face but not by voice. Topographical disorientation, but no general cognitive impairment, language disorders, or episodic memory disorders. No defects of visual perceptual or visual–spatial abilities.
Anatomical lesions	Extensive damage in the whole right temporal lobe, starting from the temporal pole. Small low-density area in the left anterior temporal lobe.
Face recognition	(Face discrimination, familiarity, identification, and naming were separately investigated.) No defect of face discrimination was found, but face familiarity and face identification were severely impaired.
Voice recognition	Severely impaired (no distinction between different components was made).
Name recognition	Identification moderately impaired.
2. KS	([66])
Diagnosis	Right temporal lobe lobectomy performed in 1973 for epileptic activity well lateralized in the right temporal lobe.
Main clinical data	After the right temporal lobectomy, KS progressively developed a severe loss of memory for people, concerning all modalities of person recognition. In response to a subjective memory questionnaire, she rated her memory as very poor on anything that had to do with people (remembering faces, voices, and proper names). Her memory problems extended to famous animals, buildings, and product names, but not to autobiographical memory.
Anatomical lesions	Removal of the anterior 6.5 cm of the right temporal lobe, including T1, the hippocampus, and the amygdala.
Face recognition	Face recognition: (face familiarity and identification were taken separately into account) she obtained normal scores on face discrimination but was clearly impaired on face familiarity and identification.
Voice recognition	Voice familiarity and identification were taken separately into account: severely impaired, particularly on the Famous Voices Test.
Name recognition	Name familiarity and name identification mildly impaired.
3. BD	([67])
Diagnosis	Herpes simplex encephalitis.
Main clinical data	Subjective complaints of inability to identify through face, voice, and name people other than his wife and immediate family and close friends. The patient was also unable to identify members of living categories and showed a severe topographical disorientation. No general cognitive impairment, language disorders, or visual perceptive and visual–spatial disorders, but mild memory impairment.
Anatomical lesions	Low-density area in the right temporal lobe involving posterior aspects of the superior temporal gyrus (CT scan).
Face recognition	(Face discrimination, familiarity, and identification were taken separately into account.) No defect of face matching, moderate impairment of face familiarity, and severe impairment of face identification and naming.
Voice recognition	Voice familiarity and identification were taken separately into account: he showed some covert familiarity associated with a severely impaired identification of famous voices.
Name recognition	Name familiarity and name identification mildly impaired.
4. VH	([68])
Diagnosis	Right-sided variant of semantic dementia?
Main clinical data	Progressive difficulty recognizing people by face. This difficulty was initially improved by hearing their voice, but this compensation progressively disappeared. Minor difficulty with memory. No intellectual, language, or visuo-perceptual disorder and a very mild memory deficit.
Anatomical lesions	Diffuse atrophy of the right temporal lobe, with sparing of the superior temporal gyrus and the hippocampal region (MRI).
Face recognition	Face discrimination, familiarity, and identification were taken separately into account: face discrimination was intact, but she was moderately impaired on familiarity judgement and severely impaired on face identification.
Voice recognition	Not formally tested. Initially clinically spared but worsened with the progression of the disease.
Name recognition	Name familiarity and name identification mildly impaired.
5. Maria	([69])
Diagnosis	Semantic dementia with greater right temporal lobe atrophy.
Main clinical data	Progressive difficulty recognizing familiar people by face (only initially improved by hearing their voice), followed by impaired visual recognition of common objects, anomia, and semantic paraphasias, but spared verbal semantic knowledge.
Anatomical lesions	Bilateral widening of the posterior portions of the lateral ventricles, greater on the right (MRI). Bilateral blood-flow reduction (SPECT).
Face recognition	Face discrimination, familiarity, and identification were taken separately into account: the severe face recognition deficits were mainly ‘associative’, but she was also impaired on some ‘apperceptive’ visual tasks.
Voice recognition	not formally tested, but hearing the voice of a familiar person did not improve identification. Furthermore, the patient no longer answered the telephone because she failed to recognize the callers from their voices. even if they were family members.
Name recognition	Mildly impaired. Person recognition was markedly improved by supplying the person’s proper name.
6. Emma	([70])
Diagnosis	Right-sided variant of semantic dementia?
Main clinical data	Progressive difficulty recognizing familiar people by face This defect initially improved by hearing the voice, but this compensation quickly disappeared. No general cognitive impairment, amnesia, apraxia, or visual–spatial defects.
Anatomical lesions	The right temporal lobe was more atrophic than the left one, where atrophy was still marginal. The atrophy encroached almost exclusively upon the anterior and inferior aspects of the right temporal lobe, leaving the posterior aspects virtually untouched.
Face recognition	(Face discrimination, familiarity, and identification were taken separately into account.) She obtained good results on apperceptive tasks but was severely impaired on face familiarity and identification tasks.
Voice recognition	(Voice discrimination, familiarity, and identification were taken separately into account.) The patient showed no apperceptive defects, being able to guess the speaker’s gender and approximate age but was unable to identify the voice of highly familiar speakers.
Name recognition	Identification moderately impaired.
7. XY	([71])
Diagnosis	Right-sided variant of semantic dementia.
Main clinical data	Progressive difficulty recognizing familiar people by face (improved by hearing their voice) and impaired smell recognition; some word-finding difficulty.
Anatomical lesions	Bilateral atrophy of the mesial temporal lobes, more pronounced on the right (MRI). Severe decreased perfusion in the entire right temporal lobe (SPECT).
Face recognition	(Face discrimination and identification were separately investigated.) The patient showed no apperceptive defect but was severely impaired on the identification of famous faces.
Voice recognition	Was clinically intact but had not been formally tested.
Name recognition	Was intact.
8. Patient 1 [38]	(subject 08 in [72] and subject B-AT2 in [40].)
Diagnosis	Severe closed head injury and right temporal lobe resection.
Main clinical data	The patient could not recognize familiar faces but has had no visual field defects and no symptoms of topographagnosia.
Anatomical lesions	Bilateral anterior temporal damage, involving more of the right than the left hemisphere and sparing the lingual and fusiform gyri bilaterally.
Face recognition	(Face discrimination, familiarity, and identification were separately investigated.) The patient showed moderate defects of face discrimination and severe defects of face familiarity and identification.
Voice recognition	(Voice familiarity and identification were taken separately into account.) Voice discrimination was preserved, whereas voice recognition was impaired.
Name recognition	Not investigated.
9. CO	([73]).
Diagnosis	Right-sided variant of semantic dementia.
Main clinical data	Progressive difficulty recognizing familiar people by face, improved by hearing their voice No general cognitive impairment or disorders of language, episodic memory, executive functions, visual perception, or visual–spatial abilities.
Anatomical lesions	Atrophy prevalent in the antero-inferior parts of the right temporal lobe (MRI), with severe hypoperfusion restricted to the anterior parts of the right temporal lobe (SPECT).
Face recognition	(Face discrimination, familiarity, and identification were taken separately into account.) CO showed no defects of face discrimination but a moderate impairment on face familiarity and a severe impairment on face identification and naming.
Voice recognition	(Voice familiarity and identification were taken separately into account.) Voice recognition was as impaired as face recognition.
Name recognition	Normal.
10. FG	([74]).
Diagnosis	Right-sided variant of semantic dementia.
Main clinical data	Progressive difficulty recognizing familiar people by face (improved by hearing the voice). Along with his face-recognition deficit, he also exhibited mild visual agnosia and mild episodic memory, visuo-perceptual, and naming difficulties.
Anatomical lesions	Bilateral atrophy of the right fusiform gyrus and parahippocampal cortex, with relative sparing of the temporo-polar cortex. The extent of the atrophy was far more extensive in the right than in the left hemisphere.
Face recognition	Face configurational processing, familiarity, and identification were taken separately into account: A mild impairment was found on face configurational processing, whereas face familiarity was moderately impaired, and face identification and naming were severely impaired.
Voice recognition	Clinically intact but not formally tested.
Name recognition	Normal.
11. JT	([75]).
Diagnosis	Behavioral variant of FTD with prevalence of right temporal lobe atrophy.
Main clinical data	Progressive change in personality, with loss of empathy and unconcern about others, followed by difficulty in recognizing familiar people through all modalities and food items. Deterioration of semantic memory extended to the recognition of words and common objects and was accompanied by a tendency to orally examine all objects repeatedly and indiscriminately.
Anatomical lesions	Significant atrophy in the right temporal pole and right amygdala/anterior hippocampal complex extending to the right insula. Bilateral atrophy of the collateral sulcus–fusiform gyrus.
Face recognition	No difficulty with face discrimination but moderate impairment of face familiarity and severe impairment of face identification and naming.
Voice recognition	(Not formally tested but severely impaired). Face recognition was not improved by hearing the person’s voice.
Name recognition	Identification severely impaired.
12. MT	([76]).
Diagnosis	Semantic dementia with more pronounced right temporal lobe atrophy.
Main clinical data	Progressive inability to learn the faces of recent acquaintances (of which the patient was unaware). She presented deficits in recognizing faces of famous people and family members but remained able to recognize familiar people by their voice. No general cognitive deterioration or disorders of language, memory, visual perception, or visual–spatial abilities.
Anatomical lesions	Bilateral atrophy of the anterior temporal lobes greater on the right side.
Face recognition	(Face familiarity and identification were taken separately into account.) The patient was moderately impaired on face familiarity, identification, and naming.
Voice recognition	(Familiarity and identification of well-known voices.) MT was able to correctly discriminate between the familiar and unknown people and was able to identify her close acquaintances.
Name recognition	Normal.
13. CD	([77]).
Diagnosis	Frontotemporal dementia with prevalence of the right temporal lobe atrophy
Main clinical data	Progressive personality change and difficulty recognizing familiar people. No difference was noted between the different modalities of person recognition. Normal results were initially obtained on memory, attention, and visual–spatial tasks, but behavioral disorders and recognition defects rapidly worsened in the following months and were followed by stereotypic behaviors, with echolalia and palilalia, and by the development of a motor neuron disease.
Anatomical lesions	Bilateral atrophy of the ventral and mesial parts of the temporal lobes, more pronounced on the right side (MRI), with extensive hypoperfusion affecting ventral and dorsal parts of the right frontal and temporal lobes (SPECT).
Face recognition	(Face discrimination, familiarity, and identification were taken separately into account.) Not impaired on face discrimination but impaired on face familiarity and face identification.
Voice recognition	(Voice familiarity and identification were taken separately into account.) Severely impaired on voice familiarity and identification.
Name recognition	Severely impaired. No difference was found in the severity of people recognition disorders tested through face, voice, and verbal definition modalities.
14. RA-T2	([78]; [40])
Diagnosis	Herpes simplex encephalitis.
Main clinical data	Thirty-year-old left-handed female, who had herpes simplex encephalitis 5 years prior to testing, leaving her with right anterior temporal damage. Since recovery, she has had trouble recognizing faces, relying on body habitus, gait, and voice cues. She has mild problems with her memory but continues to function well in her work at a bank.
Anatomical lesions	Right anterior temporal lesion with preservation in both hemispheres of the fusiform and occipital face areas and the superior temporal sulci.
Face recognition	(Face discrimination, familiarity, and identification were separately investigated.) No defect of face discrimination (apart from a mild impairment in discriminating changes in identity when expression also varied, suggesting some difficulty with invariant representations) but severe disorders of face familiarity and identification.
Voice recognition	(Voice familiarity and identification were taken separately into account.) No disorders of voice discrimination but severe defects of voice familiarity and identification.
Name recognition	Not investigated.
15. B-AT1	([78]; [21])
Diagnosis	Herpes simplex encephalitis
Main clinical data	A 24-year-old right-handed male who, 3 years prior to testing, suffered from herpes simplex encephalitis. Since recovery, he has had extreme difficulty in recognizing faces, particularly with learning new faces, though he can recognize some family members. Furthermore, he showed poor facial recognition for family members of whom he could provide reliable semantic information. Episodic memory and mental functioning were unaffected, allowing him to attend college and hold full-time employment.
Anatomical lesions	Bilateral anterior temporal lesions with preservation in both hemispheres of the fusiform and occipital face areas and the superior temporal sulci.
Face recognition	(Face discrimination, familiarity, and identification were separately investigated.) The patient did well on a perceptual test of face matching but showed severe defects of face familiarity identification.
Voice recognition	(Voice familiarity and identification were taken separately into account.) The patient showed mild disorders of voice discrimination but severe defects of voice familiarity and identification.
Name recognition	Not investigated.
16. MD	([79])
Diagnosis	Right temporal variant of frontotemporal dementia.
Main clinical data	Slow progressive deterioration of the ability to recognize familiar persons by face, associated with difficulties in finding and understanding names of persons and common objects. These difficulties were associated with a progressive change in behavior and personality and with a tendency towards stereotypical and compulsive behaviors, narrowed preoccupations, and irritability.
Anatomical lesions	Clear atrophy of the anterior parts of the temporal lobes with right lateral dominance (MRI) with hypoperfusion restricted to the anterior parts of the right temporal lobe and to the right frontal lobe, insula, and parahippocampal gyrus (PET).
Face recognition	(Face discrimination, familiarity, and identification were taken separately into account.) No defects of face discrimination. Moderately impaired on face familiarity and severely impaired on face identification and naming.
Voice recognition	(Voice familiarity and identification were taken separately into account.) The patient was severely impaired in both familiarity and identification of the voices of her relatives.
Name recognition	Identification severely impaired.
17. QR	([80]).
Diagnosis	Behavioral variant frontotemporal dementia.
Main clinical data	Insidiously progressive behavioral decline, with impassivity and loss of empathy. Impaired voice recognition with relatively spared recognition of faces. Mild general cognitive impairment, with anomia and impaired lexical comprehension.
Anatomical lesions	Bilateral frontotemporal atrophy, greater in the frontal lobes and in the right anterior temporal lobe, extending posteriorly and including the superior temporal sulcus
Face recognition	(Face discrimination, familiarity, and identification were separately investigated.) Face discrimination was normal, face familiarity was moderately impaired (due to an inflated false alarm rate), and face identification and face naming were more clearly impaired.
Voice recognition	(Voice discrimination, familiarity, and identification were separately investigated.) Voice discrimination was normal, but voice familiarity, identification, and naming were severely impaired. QR demonstrated a clear superiority for recognition of faces versus voices.
Name recognition	(Familiarity) mildly impaired,
18. KL	([80]).
Diagnosis	Temporal variant frontotemporal lobar degeneration with right temporal lobe atrophy.
Main clinical data	Progressive difficulty in recognizing acquaintances, followed by progressive difficulty with word finding and topographical memory. Mild anomia.
Anatomical lesions	Bilateral predominantly anterior temporal lobe atrophy, more marked on the right side and in the inferior temporal cortices, including the fusiform gyrus.
Face recognition	(Face discrimination, familiarity, and identification were taken separately into account.) Face discrimination was normal, face familiarity was moderately impaired, and face identification and naming were severely impaired.
Voice recognition	(Voice discrimination, familiarity, and identification were separately investigated.) Voice discrimination was normal, voice familiarity was moderately impaired, and voice identification and naming were severely impaired.
Name recognition	Familiarity moderately impaired.
19. B-ATOT2	([40]; [81])
Diagnosis	Herpes simplex encephalitis.
Main clinical data	After the HSE episode, the patient had difficulty reading and spelling, which improved, and more enduring topographagnosic symptoms. She showed familiar people recognition disorders and obtained borderline or mildly abnormal results on visual perceptual, attentional, and memory tests.
Anatomical lesions	The MRI showed bilateral fusiform lesions and right-sided lesions of the lateral temporo-occipital and medial occipito-parietal cortex and of the medial aspect of the temporal pole and inferior temporal cortex.
Face recognition	(Face discrimination, familiarity, and memory were separately investigated.) The patient showed some face configurational disorders and severe defects in face familiarity and identification.
Voice recognition	(Voice familiarity and identification were taken separately into account.) Both voice discrimination and voice recognition were impaired.
Name recognition	Not investigated.
20. RA-T3	([40]; [82]).
Diagnosis	Herpes simplex encephalitis.
Main clinical data	After the HSE episode, the patient showed disorders of familiar face identification but obtained normal or borderline results on visual perceptual, attentional, and memory tests.
Anatomical lesions	The MRI showed an important lesion in the right anterior temporal lobe with preservation of the fusiform and occipital face areas and the superior temporal sulci in both hemispheres.
Face recognition	(Face discrimination, familiarity, and identification were separately investigated.) No defect in face discrimination but severe disorders of face familiarity and identification.
Voice recognition	(Voice discrimination, familiarity, and identification were taken separately into account.) No defects in voice discrimination, familiarity, and identification were found.
Name recognition	Not investigated.
21. BD	([83])
Diagnosis	Right temporal variant of frontotemporal dementia.
Main clinical data	Insidious onset of personality changes, with apathy, loss of empathy, and ritualistic behaviors accompanied by familiar people recognition defects in a context of relatively intact cognitive functions.
Anatomical lesions	The MRI, implemented with voxel-based morphometry (VBM) and region of interest (ROI) analyses, showed a focal atrophy of the right ATL (temporo-polar cortex and anterior parts of perirhinal and entorhinal cortices).
Face recognition	(Face discrimination, familiarity, and identification were separately investigated.) The patient showed no defects of face discrimination but was moderately impaired on face familiarity, identification, and naming.
Voice recognition	(Voice discrimination, familiarity, and identification were taken separately into account. The patient showed no defects of voice discrimination but was severely impaired on voice familiarity, identification, and naming.
Name recognition	Familiarity and identification were normal. The patient obtained normal scores on people recognition and identification through personal names when written names were presented.
22. MM	([84])
Diagnosis	Ischemic stroke in right subcortical structures and right anterior temporal lobe.
Main clinical data	After fibrinolytic treatment, the patient progressively recovered from their right-sided stroke and was discharged without neurological focal signs. When he returned home, he noticed he was not able to recognize the voices of known singers but did not note problems in identifying the voices of his relatives. Probably he had never needed to identify them exclusively by their voice, because he interacted with them face-to-face. All neuropsychological domains except voice recognition were spared.
Anatomical lesions	Neuroimaging carried out by means of PET and MRI revealed two small ischemic lesions in the right subcortical region, involving lenticular and caudate nuclei and in the right temporal pole.
Face recognition	(Face discrimination, familiarity, and identification were separately investigated.) No defects of face discrimination, familiarity, identification, and naming were found.
Voice recognition	Voice discrimination, familiarity, and identification were taken separately into account. No defect was found in voice discrimination, but he was severely impaired in voice familiarity, identification, and naming.
Name recognition	Normal.

### 3.1. Analysis of Data Reported in Table 1 for Each Variable Considered in the Present Review

#### 3.1.1. Etiopathological Diagnosis

Two main groups of patients were identified from the etiopathological point of view. The first group was formed by 13 patients (4, 5, 6, 7, 9, 10, 11, 12, 13, 16, 17, 18, and 21), representing 58% of the whole sample, who showed a right frontotemporal degeneration (FTD). The second group was formed by six patients (1, 3, 14, 15, 19, 20), corresponding to 27%, who presented the after-effects of an episode of herpes simplex encephalitis (HSE). Two patients (2 and 8) had undergone an operation of right temporal lobectomy due to epileptic activity in the right temporal lobe (patient 2) and to a closed head injury (patient 8). The last patient (22) had suffered from an ischemic stroke.

#### 3.1.2. Main Clinical Data

Subjective complaints of inability to identify familiar people by face were in the foreground in 12 patients (1, 3, 4, 5, 6, 7, 8, 9, 10, 12, 14, and 18, representing 57% of the whole sample), whereas a progressive change in personality with loss of empathy had been the opening symptom in patients 11, 13, 15, 17, and 21. A loss of memory for people, concerning all modalities of person recognition, was reported in patients 2, 11, 13, and 16, and an inability to recognize the voice of known singers was reported in patient 22.

Details concerning the onset and evolution of the different modalities of person recognition disorder had been included only for 17 of the clinical cases reported in Table 1, because these data had not been reported for patients 8, 14, 15, 19, and 21. Within the 17 patients for whom this information was available, all modalities had been jointly affected since the onset of the symptomatology in 7 patients (2, 3, 11, 13, 16, 18, and 19); the face had been the first affected modality in 9 patients (1, 4, 5, 6, 7, 9, 10, 12, and 20), and the voice modality had been the first (and unique) modality affected in patient 22. Furthermore, in patients 4, 5, 6, 7, 9, and 10, the difficulty of recognizing familiar people by face was initially improved by hearing their voice, even if this compensation progressively disappeared in patients 4, 5, 6, and 9.

#### 3.1.3. Neuroanatomical Data

Only in patients 2, 14, 20, and 21 were the anatomical lesions restricted to the right ATL, because in most patients the lesions extended to posterior (patients 3 and 4) or subcortical (patient 22) right hemisphere structures and (to a lesser extent) also to the left hemisphere in 15 patients (1, 5, 6, 7, 8, 9, 10, 11, 12, 13, 15, 16, 17, 18, 19), who represented the majority (52%) of the whole sample.

#### 3.1.4. Face Recognition

Both the apperceptive and the associative disorders of face recognition had been formally investigated in all 22 patients included in the present review. An associative disorder, involving face familiarity and face identification, had been documented in all of them, with the exception of patient 22, who presented a pure form of associative phonagnosia. An associative form of prosopagnosia was, therefore, present in 95% of patients included in our sample. In patients 5, 10, 14, and 19, mild features of apperceptive prosopagnosia had been found in association with face familiarity and face identification disorders.

#### 3.1.5. Voice Recognition

A formal assessment of voice recognition was not available for patients 4, 5, 7, 10, and 11. Furthermore, apperceptive and associative components of phonagnosia had not been separately investigated in patient 1. Within these six patients (representing 27% of the whole sample), the voice had been considered as clinically intact in patients 7 and 10 and as severely impaired in patients 1, 5, and 11. A severe impairment in the identification of familiar voices had been observed in the other 16 patients. An associative form of phonagnosia was, therefore, present in 88% of patients included in our sample, and a mild defect of voice discrimination had been reported in patients 15 and 19.

#### 3.1.6. Name Recognition

This ability was assessed at the level of name familiarity and/or of person identification from name in 17 patients (1, 2, 3, 4, 5, 6, 7, 9, 10, 11, 12, 13, 16, 17, 18, 21, and 22), representing 70% of the whole sample. It was found severely impaired in three patients (11, 13, and 16), moderately impaired in three patients (1, 6, and 18), mildly impaired in five patients (2, 3, 4, 5, and 17), and intact in six patients (7, 9, 10, 12, 21, and 22). Name recognition disorders were, therefore, present in 11/17 (65% included in our sample). When the severity of person recognition defects was compared on similar tasks across verbal (name) and non-verbal (face and voice) modalities, this severity was similar only in patients 11 and 13, whereas it was greater for face and voice than for name in all the other patients.

#### 3.1.7. Associations and Dissociations Between Face, Voice, and Name Recognition Disorders

Only three ‘pure forms of associative prosopagnosia’ (patients 7, 12, and 20) and one ‘pure form of associative phonagnosia’ (patient 22) had been documented in this review, whereas all verbal and non-verbal modalities of people recognition had shown some level of impairment in 11 patients (1, 2, 3, 4, 5, 6, 10, 13, 16, 17, and 18), representing one half (50%) of the whole sample. These defects were usually greater in the face and voice than in the name recognition modality. Name recognition disorders had not been taken into account in five patients (8, 14, 15, 19, and 20), and a concomitance of associative forms of prosopagnosia and phonagnosia had been observed in four of them (8, 14, 15, and 19). The sequence of presentation of these disorders was not randomly distributed in 13 patients (4, 5, 6, 7, 9, 10, 11, 12, 13, 16, 17, 18, and 21), affected by a progressive form of FTD, who represented the majority (62%) of the whole sample, because defects in face recognition appeared first and were for some time compensated by hearing the voice of persons not recognized by face in patients 4, 5, 6, 7, 9, 10, 12, and 16, whereas voice recognition disorders appeared first only in patient 17.

We can, therefore, conclude that (a) in patients included in the present review, the associative forms of prosopagnosia and phonagnosia were more frequent than the name recognition disorders; (b) the non-verbal disorders of familiar people recognition tended to co-occur, but face recognition disorders were more frequent and severe, tended to appear first, and were for some time compensated by hearing the voice of persons not recognized by face; and (c) in less frequent instances, it was possible to document few pure forms of associative prosopagnosia and a single pure form of associative phonagnosia.

## 4. Discussion

The findings of this review allow us to revisit the long-standing debate about whether associative prosopagnosia and phonagnosia reflect modality-specific disruptions or a shared multimodal person-recognition system. Below, we interpret the data in light of (a) the rarity of ‘pure’ cases, (b) the high frequency of multimodal impairments, and (c) the neuroanatomical patterns observed.

The first point that I intended to clarify in this review consisted of assessing if in the surveyed sample were present ‘pure’ (i.e., modality-specific) associative forms of prosopagnosia and of phonagnosia and if these ‘pure’ forms constituted a sizeable part, or a small minority, of the total number of patients included in this review. Modality-specific defects of face identification could, indeed, suggest that associative forms of prosopagnosia are due to disruption of the last steps of the process of face recognition, whereas the concomitance of a voice or name identification disturbance could lead to considering associative prosopagnosia as a simple component of a multimodal semantic person recognition disorder. From a theoretical point of view, it could be suggested that modality-specific forms of face and voice recognition disorders are due to disruption of ventral and dorsal pathways, connecting the ‘core’ components of the face- and voice-processing systems (located in posterior occipital and temporal regions and in the posterior superior temporal sulcus) to the ventral and dorsal parts of the ATLs (e.g., [85,86,87,88]). On the basis of other neuroanatomical models (e.g., [89,90]), it could also be suggested that visual (face) data, processed in the ventral ATL, and auditory (voice) data, processed in the dorsal ATL, may converge in the right mesial perirhinal and entorhinal cortices and in the right temporal pole, creating a multimodal non-verbal representation of familiar people. Finally, the basis of still other models, such as the abstract and amodal model of the ‘Semantic Hub’ (e.g., [91,92,93]), it could be suggested that all semantic aspects of familiar people may be represented in an amodal (verbal and pictorial) form in both ATLs. These neuroanatomical models could, therefore, be used as a reference frame for our discussion. This could, however, be inappropriate, because, due to the nature of our study, detailed neuroanatomical data were not available for many patients included in our review. The problem of the neuroanatomical correlates was, therefore, taken into account only for patients 21 (BD) and 22 (MM), who showed highly specific disorders of familiar people recognition and had been exhaustively examined in our laboratories. In all the other patients a descriptive approach to the characteristics shown by patients with modality-specific and multimodal person recognition disorders was used to see if a discussion of these data could help to clarify the mechanism underlying these person recognition disorders.

### 4.1. Description of the Characteristics Shown by Patients with Modality-Specific and Multimodal Forms of Person Recognition Disorders

In our sample the characteristics of ‘pure’ and ‘multimodal’ variants of associative prosopagnosia were the following:

1. **A ‘pure’ (modality-specific) variant of prosopagnosia** had been observed in patients 7, 12, and 20. Two of these patients (7 and 12) were affected by relatively mild right variants of FTD, whereas patient 20 had shown disorders of familiar face identification after an episode of herpes simplex encephalitis. In this last patient, the lesion was strongly circumscribed to the right ATL, whereas in patients 7 and 12, the ATL atrophy was bilateral, though greater on the right side. The existence of pure ‘associative’ forms of people recognition disorders had been confirmed in our review by the report of a case of ‘pure associative phonagnosia’ in a patient (22) who had suffered from an ischemic stroke in right subcortical structures and right ATL.

2. On the other hand, data documenting **a multimodal disruption of personal semantics in all recognition modalities** had been found in at least six patients (1, 6, 11, 13, 16, and 18), who had shown clear evidence of associative impairment in both the verbal and non-verbal modalities of people recognition. The multimodal nature of this person recognition disorder (and the widespread underlying brain damage) had also been supported by the fact that in two of them (patients 11 and 13) the severity of person recognition defects was comparable across modalities. Furthermore, in patient 11, recognition disorders extended from person identification to recognition of food items, whereas in patient 13, person recognition disorders had been followed by stereotypic behaviors, with echolalia and palilalia, and by the development of a motor neuron disease.

3. Finally, **a multimodal person recognition disorder, restricted to the non-verbal (face and voice) modalities,** had been observed in patient 21, who had shown clear associative forms of prosopagnosia and phonagnosia in a context of intact name recognition abilities. Furthermore, at variance with Liu et al.’s [40] observation of a bilateral ATL damage in their two subjects showing a co-occurrence of associative forms of prosopagnosia and phonagnosia, in this patient, the focal atrophy was restricted to the right ATL (temporo-polar cortex and anterior parts of perirhinal and entorhinal cortices). Even if it is impossible to claim that in this case with focal degeneration, the neuropathology was strictly limited to the right ATL, the implementation of the MRI study with VBM and ROI analyses allowed for excluding the presence of a significant left-sided involvement. Furthermore, in this case, the anatomical locus of the lesion was not consistent with the hypothesis of an association between independent face and voice recognition disorders. As a matter of fact, in the case of pure phonagnosia (patient 22) of this review, the lesion affected a dorsolateral part of the ATL, which, according to Barton and Corrow (2016) [45], should result in a selective or prevalent voice recognition deficit, whereas in case 21, with face and voice recognition disorders, the lesion mainly affected the medial structures of the ATL, namely the anterior parts of the perirhinal and entorhinal cortices. Since these are areas of multimodal integration, strictly linked to the hippocampus [22], it is not likely that this lesion may have also affected the (dorsolateral) structures underpinning the highest levels of voice processing. It seems more reasonable to assume, in agreement with the model proposed by Collins and Olson (2014) [22], that the lesion of the right mesial perirhinal and entorhinal cortices may have directly caused a multimodal non-verbal form of people recognition disorder.

### 4.2. Other Reasons Suggesing That Some Associative Forms of Prosopagnosia and Phonagnosia Might, Indeed, Be Fragments of a More General Multimodal Semantic Disorder

In addition to the identification of a patient with a multimodal person recognition disorder restricted to the non-verbal (face and voice) modalities, two other aspects of the relations between the associative forms of prosopagnosia and phonagnosia observed in the present review could support the existence of multimodal forms of person recognition disorder. The first is that this co-occurrence was very frequent in patients who had not shown a ‘pure form’ of prosopagnosia or of phonagnosia (i.e., in patients 1, 2, 3, 4, 5, 6, 8, 9, 10, 11, 13, 14, 15, 16, 18, 19, and 21). The second aspect concerns the chronological order of presentation of face and voice recognition disorders, because if we assume that the association between these disorders is due to the effect of independent lesions, then the order of presentation should be random in an unselected group of patients. On the contrary, in patients included in the present review, face recognition disorders tended clearly to appear first, because in seven of the eight patients in whom this order of presentation had been clinically recorded (i.e., in patients 4, 5, 6, 7, 9, 10, and 12), face recognition defects had been the first symptom. Furthermore, this symptom was initially improved by hearing the voice of the known person, whereas only in patient 17 voice recognition defects had been reported first, with relative sparing of face recognition abilities. This asymmetry in the order of presentation of face and voice recognition disorders must, however, be considered with caution, because in normal subjects, both familiarity judgment and retrieval of semantic information are more difficult from voices than from faces of celebrities (e.g., [94,95]).

More in general, if we try to reframe the contrast between ‘pure’ (modality-specific) and ’multimodal’ patients in neuroanatomical terms, we could suggest that pure deficits occur primarily when focal damage is restricted to the ventral or dorsal ATL subregions supporting modality-specific person-recognition codes, whereas multimodal impairments emerge when lesions involve integrative ATL hubs or extend beyond the ATL. In this case, the multimodal impairment could be restricted to the non-verbal face and voice modalities when the lesion involves only the right mesial perirhinal and entorhinal cortices and the right temporal pole, whereas it could involve verbal and non-verbal recognition modalities when the lesion extends from the right to the left ATL.

## 5. Concluding Remarks

Data reported in this review suggest that the associative forms of face and voice recognition disorders can be due to different mechanisms and be underpinned by different anatomical lesions. In a few ‘pure’ patients with lesions restricted to the right ATL, they can be due to the isolated disruption of the highest levels of modality-specific recognition systems concerning the identification of familiar face **or** voice stimuli. In many more patients with larger lesions extending from the ATLs to more posterior and superior portions of the right temporal lobes, these disorders can be due to the joint disruption of modality-specific recognition systems concerning the identification of familiar face **and** voice stimuli. In patients with bilateral lesions, involving both the right and the left ATL, it is possible to observe more complex forms of familiar people recognition disorders, involving both verbal (name) and non-verbal (face and voice) stimuli. Finally, in a few patients with lesions restricted to the right temporo-polar and anterior parts of the perirhinal and entorhinal cortices, it is possible to observe a co-occurrence of associative forms of prosopagnosia and phonagnosia that could be due to a multimodal non-verbal form of familiar people recognition disorder, rather than to the co-occurrence of independent associative deficits of face and voice identification. These different anatomo-clinical interpretations must, in any case, be consistent with functional neuroanatomical models (e.g., [22,88,89,90]), which maintain that the associative forms of face and voice recognition could be processed in distinct portions of the ATLs. Facial identity could, indeed, be processed in the ventral ATL, whereas vocal identity could be processed by the dorsal ATL, and only the temporal pole could be situated at the intersection of high-order visual/face and auditory/voice areas with areas involved in social–emotional processes. It is, therefore, interesting to note that in patient BD [83], affected by an initial form of right temporal variant of frontotemporal dementia, there was a strong consistency between clinical and neuroanatomical data. In fact, in this patient, who showed no defects of face and voice discrimination and of name recognition but was impaired on face and voice familiarity identification and naming tasks, the MRI, implemented with VBM and ROI analyses, showed a focal atrophy of the right temporo-polar cortex and of the anterior parts of perirhinal and entorhinal cortices.

More in general, the present review aligns with models proposing a graded specialization within the ATL rather than strict modularity. Pure associative prosopagnosia and phonagnosia emerged when lesions were limited to dorsal/ventral ATL subregions, consistent with modality-specific processing streams. Conversely, multimodal non-verbal person recognition disorders appeared when medial ATL subregions (perirhinal and entorhinal cortices) and temporal poles were affected, supporting their role as high-level integrative person identity hubs.

## Data Availability

No new data were created or analyzed in this study.
